# Succinylation and redox status in cancer cells

**DOI:** 10.3389/fonc.2022.1081712

**Published:** 2022-12-20

**Authors:** Xiaofeng Dai, Yanyan Zhou, Fei Han, Jitian Li

**Affiliations:** ^1^ Wuxi School of Medicine, Jiangnan University, Wuxi, China; ^2^ Henan Luoyang Orthopedic Hospital (Henan Provincial Orthopedic Hospital), Zhengzhou, China

**Keywords:** succinylation, cancer, redox status, cell metabolism, cold atmospheric plasma

## Abstract

Succinylation is a post-translational modification (PTM) event that associates metabolic reprogramming with various pathological disorders including cancers *via* transferring a succinyl group to a residue of the target protein in an enzymic or non-enzymic manner. With our incremental knowledge on the roles of PTM played in tumor initiation and progression, relatively little has been focused on succinylation and its clinical implications. By delineating the associations of succinylation with cancer hallmarks, we identify the, in general, promotive roles of succinylation in manifesting cancer hallmarks, and conceptualize two working modes of succinylation in driving oncogenic signaling, i.e., *via* altering the structure and charge of target proteins towards enhanced stability and activity. We also characterize succinylation as a reflection of cellular redox homeostatic status and metabolic state, and bring forth the possible use of hyper-succinylated genome for early cancer diagnosis or disease progression indication. In addition, we propose redox modulation tools such as cold atmospheric plasma as a promising intervention approach against tumor cells and cancer stemness *via* targeting the redox homeostatic environment cells established under a pathological condition such as hypoxia. Taken together, we emphasize the central role of succinylation in bridging the gap between cellular metabolism and redox status, and its clinical relevance as a mark for cancer diagnosis as well as a target in onco-therapeutics.

## Introduction

Succinylation, a post-translational modification (PTM) process that modulates a protein’s structure by transferring a succinyl group (-CO-CH_2_-CH_2_-CO_2_H) to a residue of the target protein, is involved in many cellular processes in human life such as mitochondrial metabolism and thus plays critical roles in various diseases including cancers (1). Chemical succinylation, though typically occurring on lysine (K), can also occur on arginine (R) or histidine (H) depending on pH (2). Yet, protein succinylation is considered a PTM event *in vivo* on lysine (3).

The discovery of lysine succinylation stemmed from studies on acetylation, where a variety of new acylation forms including succinylation were identified that had substantially enriched the PTM family (4). In 1961, succinylation was first used as a method of inhibiting antibody formation to test for erythematous responses in animals sensitive to dinitrophenyl-polyline (DNP-polyline) (5). The mechanisms underlying this antibody inhibition was not elucidated until 1976, when succinylation was found to alter the electrophoretic mobility, isoionic pH and conformational shift of ovalbumin *in vitro (* 6). In 1992, the transcriptional properties of succinylated nucleosomal cores were reported to be similar to those of acetylated particles (7, 8). In 2004, lysines were found to be indispensable for the binding of the succinyl group to the target proteins (4, 9). In 2011, succinylation was revealed as a naturally occurring PTM on lysine residues in bacteria (3, 4, 10). Since then, intensive efforts had been devoted to succinylation, where proteins targetable by succinylation such as histones, specific succinylation sites, enzymes catalyzing succinylation and de-succinylation, as well as the roles of succinylation in both the physiological and pathological cell states were most frequently reported (4, 10–17). Investigations using mass spectrometry (MS)-based proteomic analysis have identified numerous lysine succinylated proteins localized in the cytoplasm, mitochondrion, and nucleus, including metabolic enzymes involved in, e.g., the metabolism of fatty acids, amino acids, and carbohydrates (18–21). Nowadays, succinylation is known as a PTM occuring extensively in both prokaryotes and eukaryotes that plays crucial roles in regulating the functionalities of various signalings and pathways (17).

Succinylation relies on succinyl-CoA to supply the succinyl groups, where the succinyl group is added to the ϵ-amino groups of protein lysine residues (15, 22). Lysine has an innate positive charge at the physiological pH that can be neutralized by PTMs such as acetylation, methylation and succinylation, leading to shifted protein structure and enzymatic properties (3, 10). The succinate group is larger, i.e., approximately 100.02 Daltons (Da) (3, 16), than other typical covalent modification groups of lysines such as acetyl (42.0106 Da) and dimethyl (28.0313 Da) (3, 23). Thus, the succinate group is bigger than the acetyl or methyl group, and thus may impose a larger force and impact to the structure and function of the target proteins (3, 4). In addition, the charge status of the lysine residue can be shifted from +1 to −1, 0 and not at all, respectively, by succinylation, acetylation and mono-methylation at the physiological pH (7.4) (4, 24), further supporting the possible stronger effect of succinylation than the other PTMs such as acetylation and methylation.

Given the recognized implications of acetylation and methylation in cancer diagnosis and therapeutics (25, 26), we are interested to delineate the roles of succinylation during tumorigenesis according to the hallmarks of cancer, its working modes in driving cancer hallmarks and the clinical relevance of succinylation, with the hope of enriching our toolboxes for cancer management and resolving issues remaining in the field of oncology.

## Mechanism and regulation of succinylation

**Figure 1:**
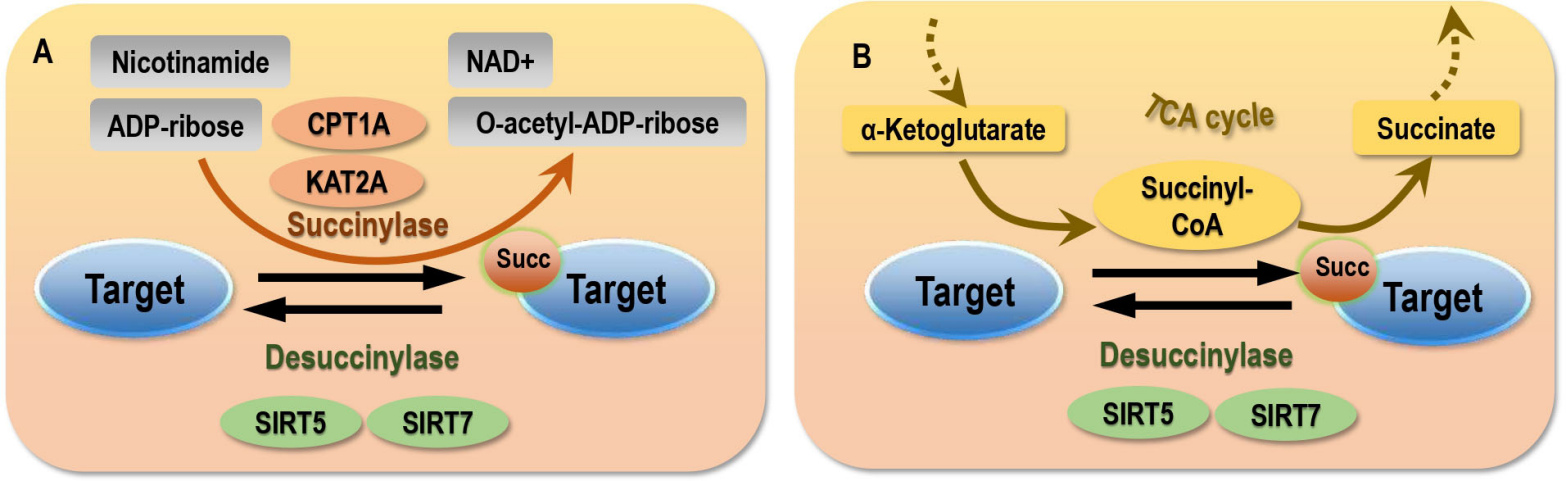
Mechanism of succinylation. **(A)** Enzymic succinylation. **(B)** Non-enzymic succinylation. Succinylases such as CPT1A and KAT2A are required in enzymic succinylation. Sufficient succinyl-CoA supply is a pre-requisit for the occurrence of non-enzymic succinylation. Desuccinylases such as SIRT5 and SIRT7 are required to erase succinylation marked *via* both enzymic and non-enzymic pathways.

Succinylation can be either enzymatically or non-enzymatically (3, 16, 19, 21, 27–29) (**Figure 1**). Both processes require succinyl-CoA that can be produced from mitochondria or peroxisomes (16, 30). While mitochondrially-derived succinyl-CoA is trapped within the matrix, peroxisome-generated succinyl-CoA finds its way to the cytosol (31–33). This makes succinylation possibly occur both on histones and non-histone proteins.

### Enzymatic succinylation

Though our knowledge on the enzymatic system of succinylation is far from complete, carnitine palmitoyltransferase1A (CPT1A) and lysine acetyltransferase 2A (KAT2A) are known succinylation ‘writers’ (19, 34), sirtuin 5 (SIRT5) and sirtuin 7 (SIRT7) are erasers of lysine succinylation (11, 35), and the yeast domain of glioma-amplified sequence 41 (GAS41) is a pH-dependent reader of lysine succinylation (36).

While KAT2A, also known as GCN5, can act as a succinyl-transferase that forms a complex with the nucleus-localized α-ketoglutarate dehydrogenase (α-KGDH) to produce succinyl-CoA (21), CPT1A, initially found responsible for catalyzing the transfer of the acyl group from coenzyme A (CoA) to L-carnitine (37), has the succinyl-transferase functionality. Specifically, 171 out of 550 lysine sites were reported succinylated on 101 out of 247 proteins in a CPT1A expression-dependent fashion without altering succinyl-CoA levels, suggestive of the succinyl-transferase role of CPT1A (19) that was independent from its other activities (38).

Mammalian cells express 7 sirtuin proteins, denoted as SIRT1-SIRT7 (39), among which SIRT5 is the sole de-succinylase so far recognized to act in all cell compartments but the de-succinylase activity of SIRT7 is limited in the nucleus (11). The activity of SIRT5 is influenced by the availability of the NAD+ (substrate), the amount of nicotinamide (product) (19), as well as interactions of SIRT5 with other regulators of cellular energy homeostasis besides sirtuins such as AMP-activated protein kinase (AMPK) and proliferator-activated receptor γ coactivator-1α (PGC-1α). Notably, PGC-1α upregulates SIRT5 expression, AMPK downregulates SIRT5 activity (40). SIRT5 is a mitochondrial protein with numerous protein targets being identified. At least 2,565 succinylation sites on 779 proteins in mammalian fibroblasts and liver tissues were found to be regulated by SIRT5 (14). In the absence of SIRT5, the rate-limiting enzyme of ketogenesis, 3-hydroxy-3-methylglutaryl-CoA synthase 2 (HMGCS2), is hyper-succinylated that leads to suppressed HMGCS2 activity and reduced ketone body production (15). SIRT5 can reverse the succinylation of pyruvate kinase M2 (PKM2) at K498, leading to increased PKM2 activity (41), de-succinylate acyl-CoA oxidase 1 (ACOX1) and isocitrate dehydrogenase 2 (IDH2) in response to oxidative damage (42, 43), and inhibit the kidney-type glutaminase (GLS) ubiquitination to regulate mitophagy and tumorigenesis (44, 45). In addition, novel targets of SIRT5 for de-succinylation have been consecutively identified such as mitochondrial uncoupling protein 1 (UCP1) in the brown fat tissues of mice (46).

### Non-enzymatic succinylation process

Several large acyl modifications including succinylation can occur predominantly by non-enzymatic mechanisms (29, 47). Succinyl-CoA maintains steady-state concentrations in the mitochondrial matrix at a low mM range (0.1-0.6 mM). Supplementing succinyl-CoA with isocitrate dehydrogenase (IDH) increased succinylation in a pH and dose-dependent manner (16, 27, 28), suggesting that protein succinylation in the mitochondria may be a chemical event aided by the alkaline pH and high concentrations of the reactive acyl-CoAs present in the mitochondrial matrix without catalytic enzymes (28, 29). In addition, it was shown that nicotinamide adenine dinucleotide phosphate (NADPH)-specific IDH mutation resulted in a 280% increase of cellular succinyl-CoA levels and mitochondrial hyper-succinylation, implicative of the driving role of succinyl-CoA and succinate on succinylation within and outside the mitochondria (13, 17).In other words, succinylation can occur if provided with sufficient succinyl-CoA supply (17).

## Succinylation and cancer hallmarks

**Figure 2:**
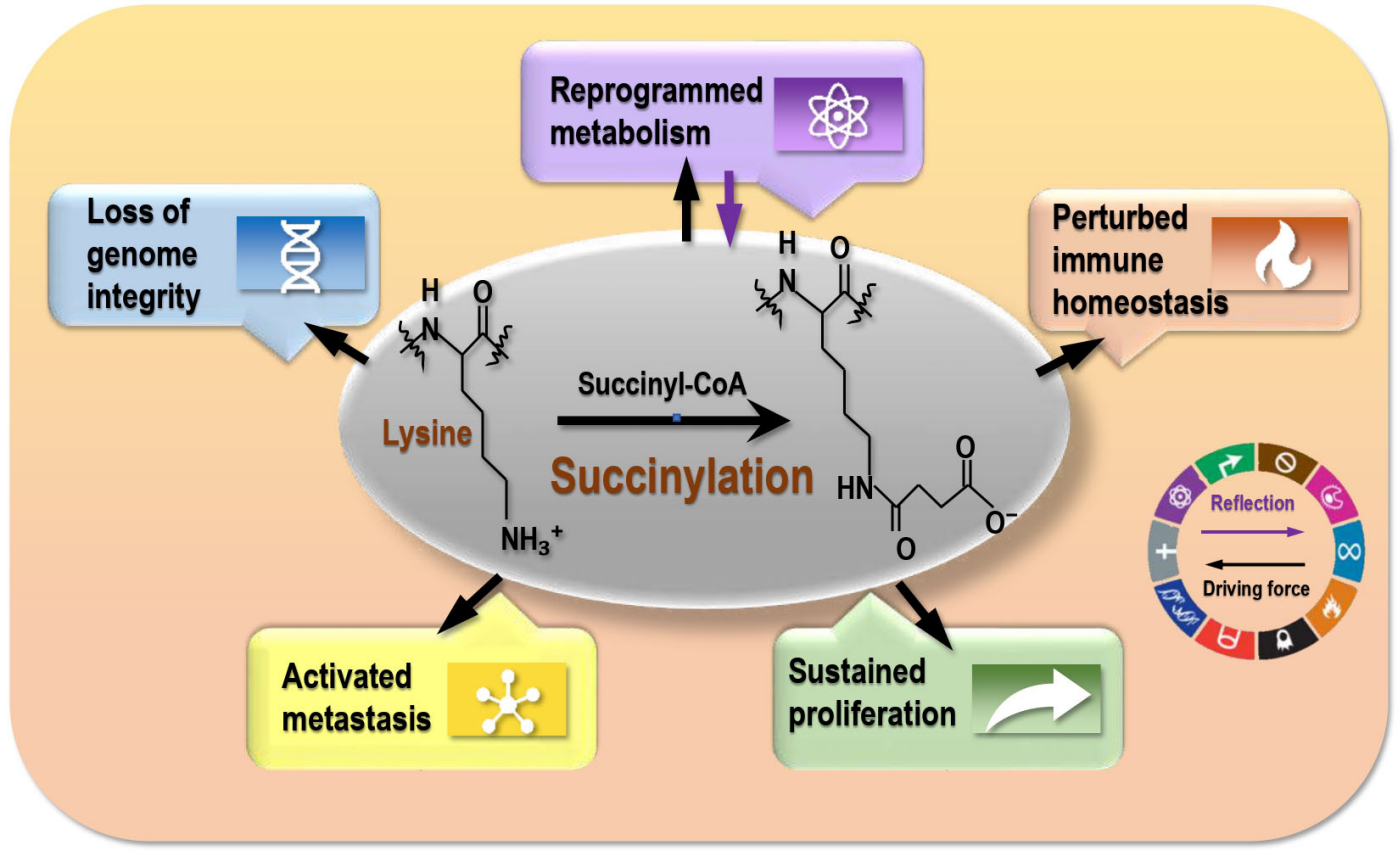
The driving role of succinylation on cancer hallmarks. Succinylation is a reflection of reprogrammed metabolism of cancer cells, and drives cancer hallmarks that are represented by ‘reprogrammed metabolism’, ‘sustained proliferation’, ‘activated metastasis’, ‘perturbed immune homeostasis’, and ‘loss of genome integrity’.

According to the five themes covered by the 10 cancer hallmarks, as established in 2000 (48), revised in 2011 (49), and updated in 2022 (50), malignant cells are featured by imbalanced cell life/death control (represented by ‘sustaining proliferative signaling’, ‘evading growth suppressors’, ‘resisting cell death’, ‘enabling replicative immortality’), metastatic transition (‘inducing angiogenesis’, ‘activating invasion and metastasis’), metabolic reprogramming (‘deregulating cellular energetics’), perturbed immune homeostasis (‘avoiding immune destruction’, ‘tumor-promoting inflammation’), and loss of genome integrity (i.e., ‘genome instability and mutation’) (25). By delineating the roles of lysine succinylation in carcinogenesis according to cancer hallmarks, we propose lysine succinylation as a reflection of disordered metabolism in histones that drives the other hallmarks of cancers (**Figure 2**).

### Hyper-succinylation accelerates glycolysis and promotes tumor growth

Succinyl-CoA, the substrate of succinylation, is produced from various metabolic processes including the tricarboxylic acid (TCA) cycle (12) that functions as the hub of energy generation and production of many biosynthetic pathway precursors. As succinyl-CoA is a metabolite generated from the TCA cycle and succinylation is widely spread among diverse mitochondrial metabolic enzymes as well as extramitochondrial cytosolic and nuclear proteins, variations on the protein lysine succinylation level may be a reflection of different cell metabolic states (51). In general, chromatin succinylation is correlated with an active transcriptional program and promotive on aerobic glycolysis and cell proliferation, and the associations between succinylation and tumorigenesis have already been established in many studies (52) such as the tumor-promotive role of phosphoglycerate mutase 1 (PGAM1) K99 succinylation in liver cancers (53) and that of fibrillin 1 (FBN1) K672 succinylation in gastric cancers (54).

Genes encoding critical enzymes of the TCA cycle especially controlling succinylation such as succinate dehydrogenase (SDH), once mutated, were reported to be associated with carcinogenesis by affecting succinylation and consequently the integrity and functionalities of the TCA cycle. Indeed, a quantitative global proteomic study reveals 161 differentially expressed lysine succinylation sites in renal cell carcinoma (RCC) tissues and significant alterations on the succinylation levels of phosphoglycerate kinase 1 (PGK1) and PKM2, suggesting the importance of lysine succinylation in energy metabolism and the driving role of glycolysis in RCC progression (55). The α-KGDH binds to KAT2A in the gene promoter regions to function as a H3K79 succinyl-transferase, the blockage or suppression of either one of which halts tumor growth (21, 34, 56). Histone acetyltransferase 1 (HAT1) is another recently identified succinyl-transferase, which promotes glycolysis and thus tumorigenesis in, e.g., human hepatoma cells and pancreatic cancer cells by enhancing the enzymic activity of PGAM1 *via* K99 succinylation (57). SIRT5, a mitochondrial NAD-dependent lysine deacylase, can suppress the biochemical activities of both the pyruvate dehydrogenase complex (PDC) and SDH as well as protect GLS from ubiquitination *via* de-succinylating GLS, which is up-regulated in transformed cells to support uncontrolled cell proliferation (14, 45). Overexpressing SIRT5 can slow the oncogenic growth of hyper-succinylated human fibrosarcoma cells that harbor IDH1 mutation, suggesting the effectiveness of hyper-succinylation inhibition in the control of hyper-succinylated tumors (13). The succinyl-CoA synthetase ADP-forming subunit β (SUCLA2) enhances kidney-type GLS K311 succinylation in response to the oxidative stress, which promotes pancreatic ductal adenocarcinoma cell survival and growth *in vivo* by enhancing the production of nicotinamide adenine dinucleotide phosphate (NADPH) and glutathione to counteract the oxidative stress (58).

### Succinylation mostly potentiates cancer metastasis

**Figure 3:**
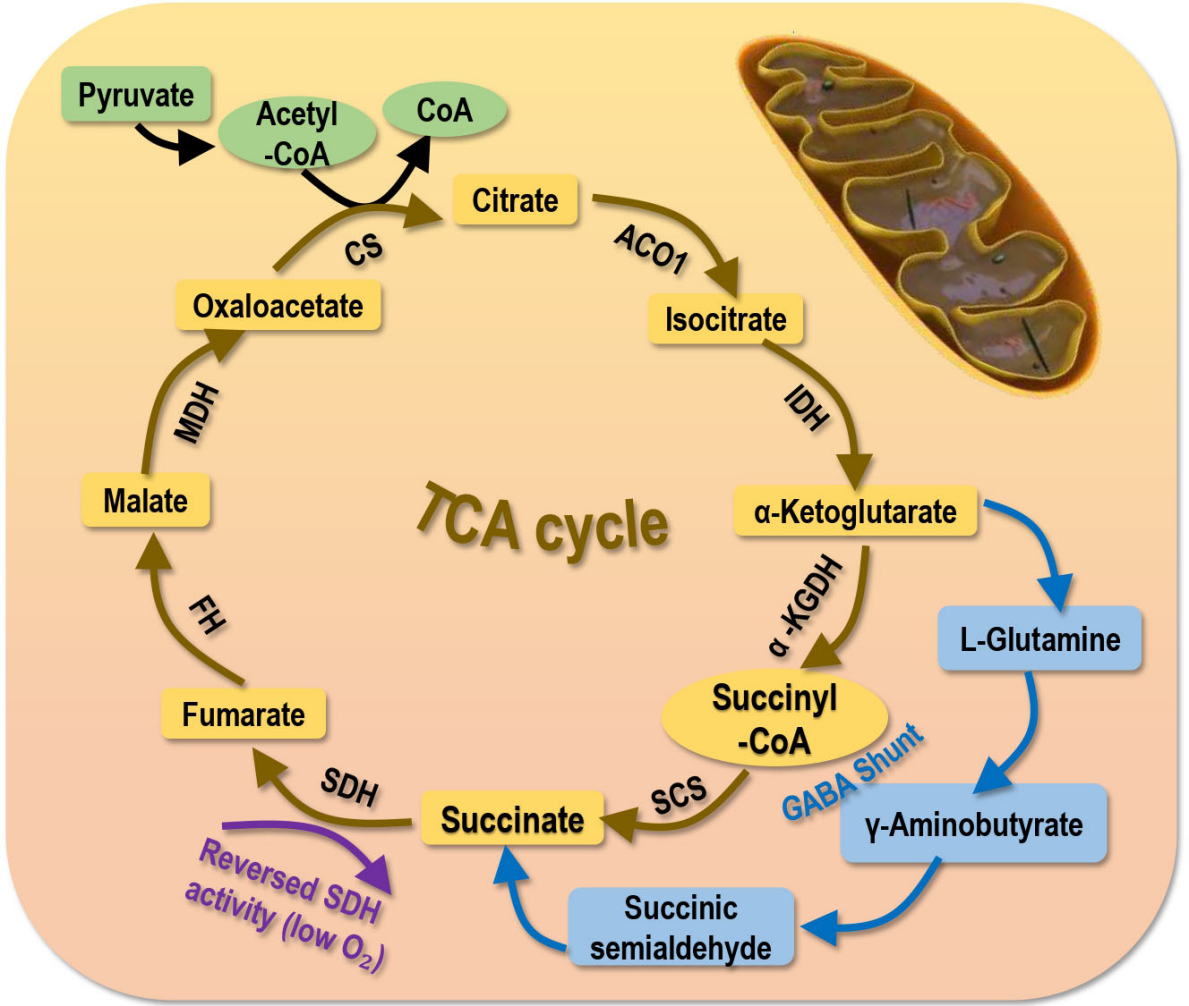
Primary sources generating succinate. Succinate is primarily generated from the tricarboxylic acid (TCA) cycle. Succinate can also be produced *via* the γ-Aminobutyric acid (GABA) shunt or under low oxygen supply that reverses the activity of SDH. SCS, succinyl-CoA synthetase; SDH, succinic dehydrogenase; a-KGDH, a-ketoglutarate dehydrogenase; IDH, isocitrate dehydrogenase; ACO1, aconitase**;** CS, citrate synthase**;** MDH, malate dehydrogenase**;** FDH, famarate dehydrogenase**;** FH, famarate hydratase.

Succinate can be generated from succinyl-CoA and oxidized to fumarate by SDH, or from other metabolic precursors such as the γ-aminobutyric acid (GABA) shunt (2, 59) (**Figure 3**). The succinyl moiety of succinylation, especially those occurring in the cytoplasm and nucleus can be originated from succinate (2). Excess succinates in the cytosol has been considered as a metabolic signature of hypoxia that is a known trigger of cancer invasion and metastasis (2, 60). Mechanically, succinates accumulate within the mitochondria as a result of reversed SDH activity and inhibited respiratory chain under low oxygen stress, and these abnormally accumulated succinates are freely transported to the cytosol *via* the dicarboxylic acid translocator in the mitochondrial inner membrane and the voltage-dependent anion channel (VDAC/porin) in the mitochondrial outer membrane to stabilize and activate hypoxia inducible factor 1α (HIF1α) (61).

Lots of evidence have associated hyper-succinylation with promoted cancer metastasis. For instance, by catalyzing H3K79 succinylation at the promoter region of tyrosine 3-monooxygenase/tryptophan 5-monooxygenase activation protein zeta (YWHAZ) to promote its expression, KAT2A reinforces the migration and invasion of many transformed cells such as human pancreatic ductal adenocarcinoma cells (62), liver cancer cells (63), and glioblastoma cells (21). CPT1A promotes gastric cancer invasion by acting as a lysine succinyl-transferase of S100 calcium binding protein A10 (S100A10) (19, 37). In addition, CPT1A succinylates lactate dehydrogenase A (LDHA) at K222 that leads to reduced LDHA degradation in gastric cancer and consequently promotes cell invasion (64). SIRT5 inhibits the activity of the oxoglutarate dehydrogenase (OGDH) complex *via* de-succinylation and thus suppresses the epithelial-to-mesenchymal transition (EMT) of gastric cancer cells (65).

Despite consecutive reports on the promotive role of succinylation on cancer progression, hypo-succinylation was revealed to be enriched in enzymes of the metabolic pathways with a promotive role on cancer migration in esophageal squamous cell cancer cells by conferring a negative regulatory role on histone methylation (66).

### Succinylation promotes tumor-associated inflammation

Succinate is a proinflammatory metabolite that was found to accumulate under certain pathophysiological situations especially inflammation. SUCNR1, being the receptor of succinate, is a plasma membrane G protein-coupled receptor (GPCP) widely expressed in many cells including those relevant to immune response such as macrophages and dendritic cells (DCs).

Succinate can tune macrophages to the M1 state that is inflammation-promotive by secreting pro-inflammatory cytokines such as interleukin-1β (IL-1β), IL-6, and interferon-γ (IFN-γ), whereas alternatively-activated M2 macrophages takes on the opposite role by expressing large amounts of IL-10 (67). Specifically, succinate is enriched in lipopolysaccharide (LPS)- or IFN-γ-treated macrophages and contribute to IL-1β transcription towards the M1 phenotype by stabilizing HIF1α as a result of PKM2 K311 succinylation, and SIRT5 prevents dextran sodium sulfate (DSS)-induced colitis by restoring PKM2 activity in a mouse model (68–70). Further, succinate produced by LPS-activated M1 macrophages can exacerbate inflammation in an autocrine and paracrine manner through enhanced IL-1β production *in vivo (* 71). On the other hand, mice lacking functional SUCNR1 are protected from acute inflammation in a colitis model, providing supportive evidence on the mediating role of the succinate-SUCNR1 axis in priming macrophages to the M1 state (72). In addition, SUCNR1 can synergize with innate Toll-like receptors to boost inflammatory responses by promoting proinflammatory cytokine secretion in DCs and myeloid cells, as well as activating T helper (Th) cells such as Th17 and attenuating T regulatory cells (71, 73–76).

### Succinylation maintains genome integrity of cancer cells

Succinylation may contribute to the maintenance of genome integrity of transformed cells and thus lead to onco-therapeutic resistance. For instance, succinylation of human flap endonuclease 1 (FEN1), a multifunctional endonuclease essential for DNA replication and repair, at K200 maintains genome stability by promoting interactions between FEN1 and the Rad9-Rad1-Hus1 complex towards a rescued DNA damage repair ability of both malignant (HeLa) and normal (HEK293T) cells; though K200 was identified as the key site for FEN1 succinylation as well as other PTM modifications such as phosphorylation and small ubiquitin like modifier 1 (SUMO-1) modification, its succinylation was found to be required for DNA damage repair, and cells deficient of this modification are sensitive to DNA-damaging agents such as hydroxyurea (77). In addition, SDH loss results in accumulated errors originated from normal DNA replication that predisposes the sensitivity of cells lacking functional SDH to genotoxic drugs such as gemcitabine and etoposide (47). On the other side, SIRT7 is associated with poly (ADP-ribose) polymerase (PARP) 1-dependent DNA damage repair system, the integrity of which is essential for cell survival in response to genotoxic stress (35). On SIRT7 depletion, deficient PARP1-dependent DNA repair leads to failed histone de-succinylation, chromatin hyper-succinylation, aberrantly elevated levels of DNA damage as represented by gamma H2A.X over-expression (52). These evidence, collectively, suggest the role of chromatin hyper-succinylation in triggering cancer cell resistance to genotoxic agents.

## Modes of succinylation driving cancer hallmarks

**Figure 4:**
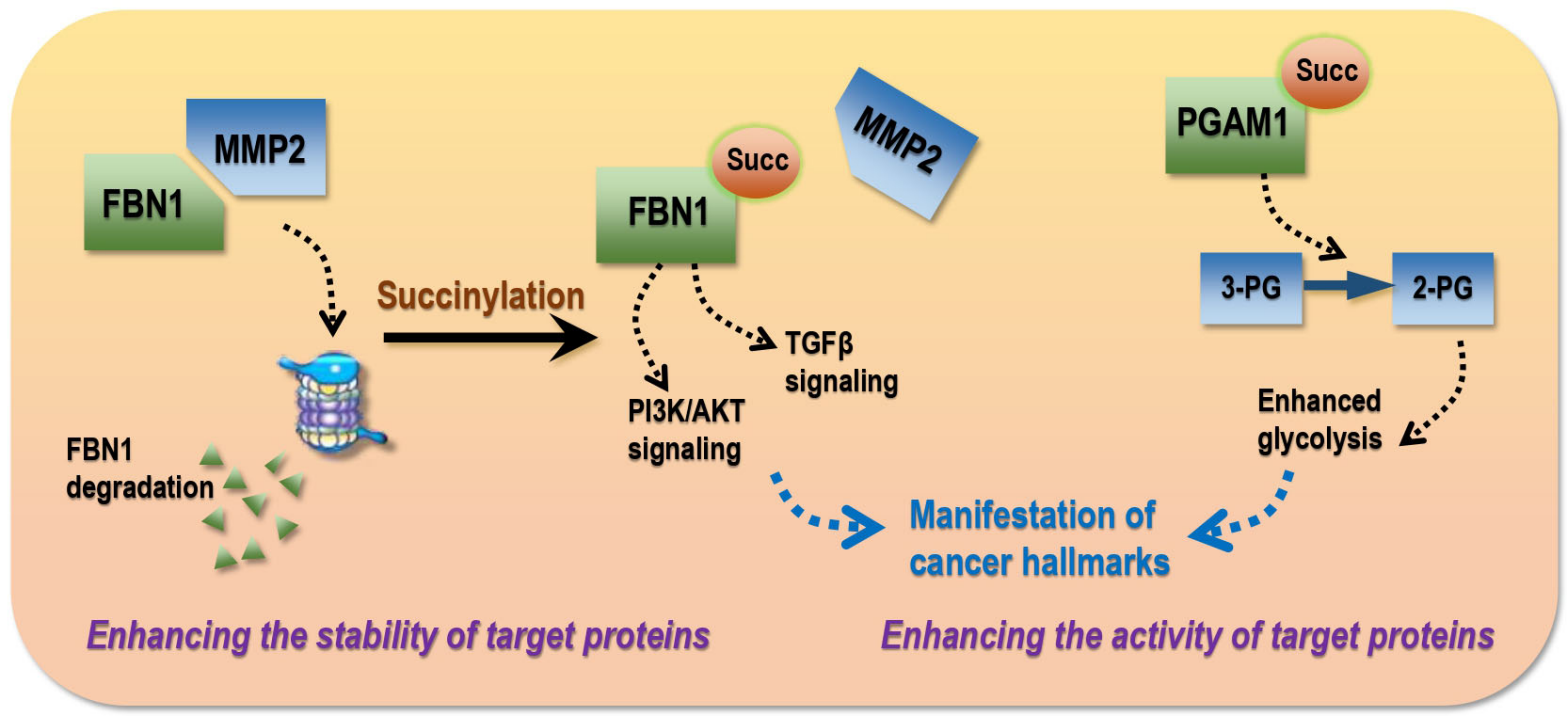
Modes of succinylation that drive the manifestation of cancer hallmarks in transformed cells. Succinylation drives cancer traits *via* two modes, i.e., enhancing the stability and/or activity of target proteins. As an example of the ‘stability’ mode, FBN1 over-expression promotes cancer progression by activating TGF-β1 and PI3K/Akt signaling, succinylation of which is widely distributed in gastric tumors that blocks its interactions with MMP2 and thus MMP2-mediated protein degradation. As an example of the ‘activity’ mode, hyper-activated PGAM1 in hepatoma cells as a result of succinylation can enhance glycolysis and thus accelerate carcinogenesis. 3-PG, 3-phosphoglycerate; 2-PG, 2-phosphoglycerate.

Most succinylations so far reported occur on proteins with oncogenic roles and promote tumor initiation and progression by increasing the (1) stability or (2) activity of these targets (**Figure 4**).

### Enhancing the stability of target proteins

Succinylation can protect target proteins from degradation by blocking protein-protein interactions (PPIs). For example, FBN1 over-expression is associated with advanced gastric malignancies that can promote cancer progression by activating TGF-β1 and PI3K/Akt signaling; succinylation of FBN1 at K672 is widely distributed in gastric tumors that blocks its interactions with matrix metallopeptidase 2 (MMP2) and thus prevents it from MMP2-mediated local degradation and collagen remodeling (54). LDHA, an enzyme controlling lactate production and the Warburg effect, is highy succinylated at K222 in gastric cancers that prevents K63-ubiquitinated LDHA from interacting with sequestosome 1 (SQSTM1) and thus reduces its lysosomal degradation (64).

### Enhancing the activity of target proteins

Succinylation can enhance the activity of target proteins. For instance, PGAM1, a key enzyme of glycolysis, accelerates carcinogenesis once hyper-activated; aspirin is capable of attenuating PGAM1 activity by removing its K99 succinylation in hepatoma cells that ultimately leads to halted tumor progression (53). Kidney-type GLS is highly represented in human pancreatic ductal adenocarcinoma specimens, the activity of which is enhanced after K311 succinylation, leading to increased glutaminolysis and elevated production of glutathione and NADPH to counteract oxidative stress for improved survival of transformed cells (58).

## Targeting succinylation *via* redox intervention as a promising onco-therapeutic strategy

**Figure 5:**
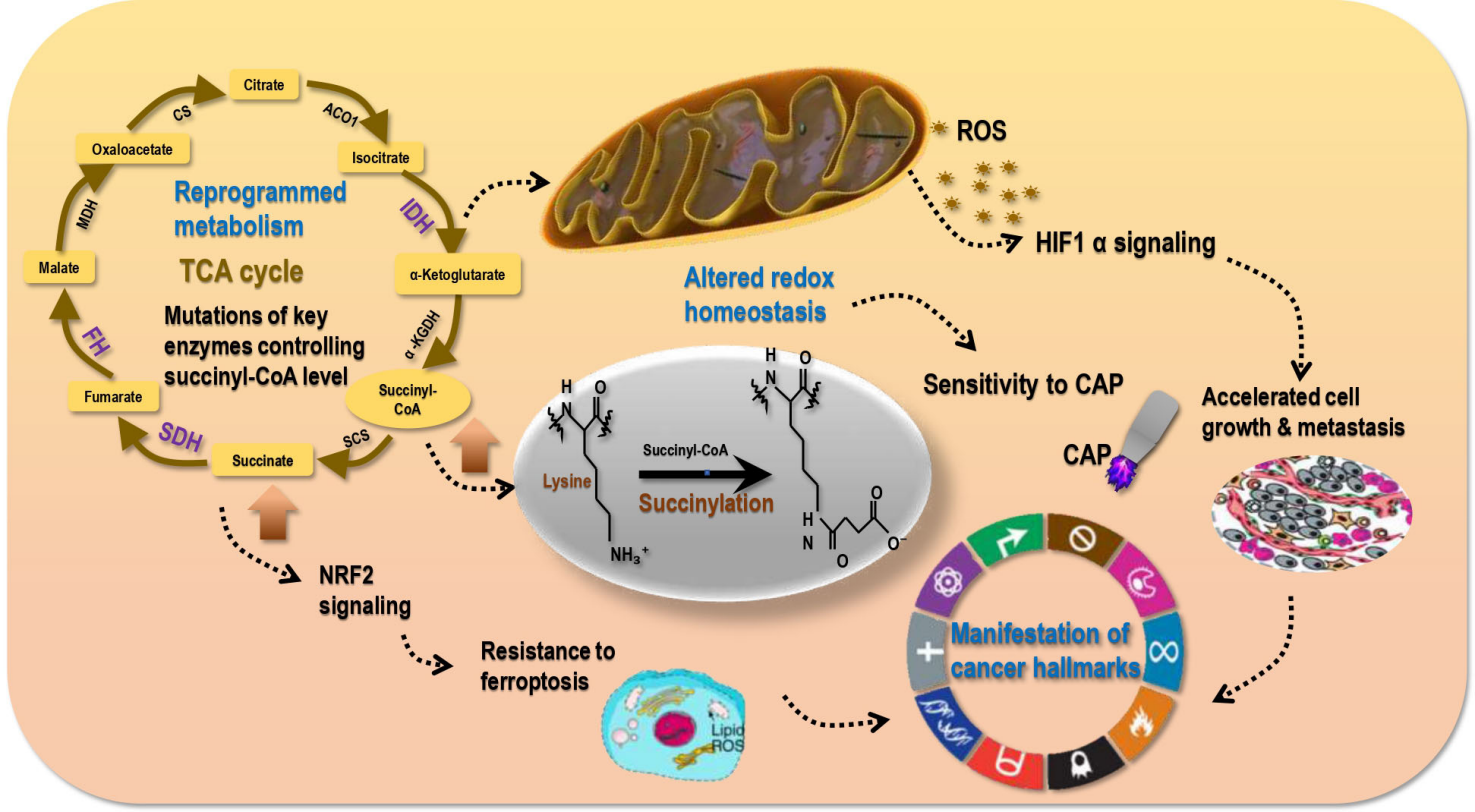
Succinylation reflects cell redox status that sensitizes tumors to redox modulation tools. Metabolism reprogramming in cancer cells as a result of, e.g., IDH, SDH, FH mutations may lead to elevated levels of succinyl-CoA and succinate. High succinyl-CoA leads to hyper-succinylation; thus, succinylation is a reflection of reprogrammed metabolism. High level of succinate results in activated NRF2 signaling that is associated with enhanced anti-oxidant ability of cancer cells (cancer stemness) and resistance of transformed cells to ferroptosis. In addition, abnormal metabolism is associated with elevated mitochondria ROS generation that is accompanied with activated HIF1α signaling and a hypoxic environment favoring cell proliferation and metastasis. These collectively result in the manifestation of cancer hallmarks and altered redox homeostasis in malignant cells. Re-established redox homeostasis in cancer cells differs from that in their healthy peers, leading to the differential sensitivities of tumor and normal cells to redox perturbation tools such as cold atmospheric plasma (CAP).

Mutations of genes encoding enzymes participating in mitochondrial metabolism such as SDH, fumarate hydratase (FH), IDH1/2 can activate the NFE2-related factor 2 (NRF2) pathway towards enhanced anti-oxidative ability and reduced ferroptosis of cancer cells by increasing succinate, fumarate, or R(-)-2-hydroxyglutarate levels that inhibit various α-ketoglutarate-dependent dioxygenases (78). In addition, altered mitochondrial metabolism can affect the cellular redox status by increasing the generation of mitochondrial reactive oxygen species (ROS) that affect the activities of transcription factors such as HIF1α (78). Therefore, succinate, being a central metabolite, not only connects several metabolic pathways, but also regulates the redox homeostasis of mitochondria and the whole cell (2); and succinylation may be a reflection of cellular metabolic and redox statuses (**Figure 5**). That is, a hyper-succinylated proteome may implicate activated aerobic glycolysis and a hypoxic cellular environment associated with stimulated HIF1α signaling that favours fast cell growth. This makes targeting succinylation *via* perturbing the redox state of transformed cells a highly promising and innovative intervention approach (**Figure 5**). Notably, non-enzymatic succinylation is largely associated with altered metabolism in cancer cells and a reflection of accelerated energy metabolism; thus, while enzymatic succinylation and its formation process may serve the purpose for cancer targeting, non-enzymatic succinylation is more feasible for cancer diagnosis.

Cold atmospheric plasma (CAP), composed of hydroxyl radical (OH·), singleton oxygen (O), superoxide (O^2-^), nitric oxide (NO), hydrogen peroxide (H_2_O_2_), ozone (O_3_), and nitrite in the form of anion or proton (OONO^-^, ONOOH) (79, 80), is a redox modulating tool and a promising onco-therapeutics with selectivity against malignant cells documented in various cancers such as melanomas (81, 82), prostate cancers (83), breast tumors (80, 84), bladder cancers (85), pancreatic cancers (86), liver carcinomas (87). By imposing a redox stress and elevating cellular ROS level, CAP may perturb HIF1α signaling and thus the redox status of transformed cells. This may either temporarily restore reprogrammed cell metabolism back to the healthy state (given that accumulated genetic mutation may consecutively drive cells towards the chaotic state once the external perturbation was removed) or, and most importantly, irreversibly trigger the cell death program such as ferroptosis by suppressing NRF2 signaling (**Figure 5**). In support of this hypothesis, CAP was reported capable of inducing ferroptosis in human lung cancer cells (88), suggesting the possible suppressive role of CAP on succinate production and succinylation due to the intrinsic connections between succinate and ferroptosis bridged by redox homeostasis (as mentioned in the beginning of this section).

At the clinical level, succinylation in general or occurring on a certain protein such as those involved in glycolysis may be employed as diagnostic markers of the progression status of malignant cells. This offers us an opportunity for early cancer diagnosis if the critical succinylation degree to which cells transit from the healthy to the transformed state was characterized. On the other hand, therapeutics relying on redox modulations such as CAP may be used as a safe yet effective tool for cancer control. As transformed cells with enhanced anti-oxidative abilities are typically more difficult to treat and exhibit traits of cancer stem cells (89), CAP may attenuate the stemness of transformed cells and represent a remedy for cancers lack of effective cure such as glioma and bone sarcoma. Given these traits, it is also promising that cancers may be eventually eradicated, to some extent, if CAP was combined with treatments targeting the bulk tumor cells and used with appropriate dosing strategy and administrating technology.

## Concluding remarks

Through delineating the relationship between succinylation and cancer hallmarks, we may centre succinylation as a PTM event reflecting the extent to which cells undergo reprogrammed metabolism that, ultimately, initiates carcinogenesis and exaggerates their uncontrolled proliferation, enhanced migrative potential, elevated tumor-associated inflammation, as well as improved genome integration and resistance to DNA-damaging therapeutics. Succinylation, adding a succinyl-group to the target protein, alters the functionalities of target proteins by changing their charges and structures. The impact of succinylation on the target proteins is expected to be larger than acetylation or methylation given the bigger mass of the succinyl-group than other common modifiers. Succinylation takes on its action by enhancing the stability or activity of proteins with oncogenic roles according to our knowledge. However, we could not exclude the possibility that succinylation may be tumor suppressive under certain circumstances. Hyper-succinylated proteome may be a reflection of abnormally accelerated metabolism, redox homeostasis under hypoxia, and programmed cell death featured by ferroptosis. Investigations on the potential of developing a gradient of succinylation indexes for cancer early diagnosis and indication of progression at different stages are of highly clinical relevance. Translating CAP into the clinics as a novel onco-therapeutic approach targeting succinylation, alone or together with the existing strategies targeting the bulk tumor cells, may bring hope to highly malignant cancers lacking effective cure and shed light on our way towards cancer eradication.

## Author contributions

XD conceptualized the insights and prepared the manuscript. YZ and FH conducted literature searching and contributed in figure preparation. All authors contributed to the article and approved the submitted version.
